# Prostatic Adenosquamous Carcinoma Metastasizing to Testis

**DOI:** 10.1100/tsw.2006.388

**Published:** 2006-10-02

**Authors:** Dilek Ertoy Baydar, Kemal Kosemehmetoglu, Bulent Akdogan, Haluk Ozen

**Affiliations:** ^1^ Department of Pathology, Hacettepe University Hospital, Sihhiye, Ankara, Turkey; ^2^ Division of Urology, Hacettepe University Hospital, Sihhiye, Ankara, Turkey

**Keywords:** adenosquamous carcinoma, metastasis, prostate cancer, sarcomatoid transformation, testis

## Abstract

Adenosquamous carcinoma of the prostate is an unusual tumor with poor prognosis. Most arise after hormonal or radiotherapy of conventional prostatic adenocarcinoma. Sarcomatous transformation in them has been reported in only a few cases. Here, we present a unique case of “de novo prostatic adenosquamous carcinoma with focal sarcomatoid areas” that showed testicular metastasis, detected after scrotal orchiectomy.

## INTRODUCTION

The incidence of squamous cell carcinoma of the prostate is less than 0.6% among prostate malignancies[1,2]. Adenosquamous carcinoma is even rarer and sarcomatous transformation in adenosquamous prostate carcinoma has been reported only in seven patients[3,4]. These tumors have poor prognosis. They are refractory to hormonal treatment and widespread metastasis at the time of diagnosis is common[5]. Our case is the first one documented with testis metastasis.

## CASE REPORT

A 56-year-old man applied to hospital with obstructive urinary symptoms. The cystoscopy showed polypoid protrusions from prostatic urethra. TURP was performed and histopathology revealed suspicious glands for prostatic carcinoma. Serum free/total PSA were found 9.02/42.27 ng/ml. Repeat TURP and sextant transrectal needle biopsies demonstrated adenosquamous carcinoma in all samples. The tumor was composed of intermingled squamous and adenoid elements with focal sarcomatoid transformation (Fig. 1). The squamous component was well differentiated with pearls and keratinization. The glands were mostly in Gleason pattern 4 with seldom grade 3 and 5 areas, and scattered "foamy gland" morphology. PSA was immunohistochemically positive in both squamous and glandular components. Although sarcomatoid areas were negative with PSA, they expressed pan-cytokeratin. Magnetic resonance imaging documented a 4.8- ×

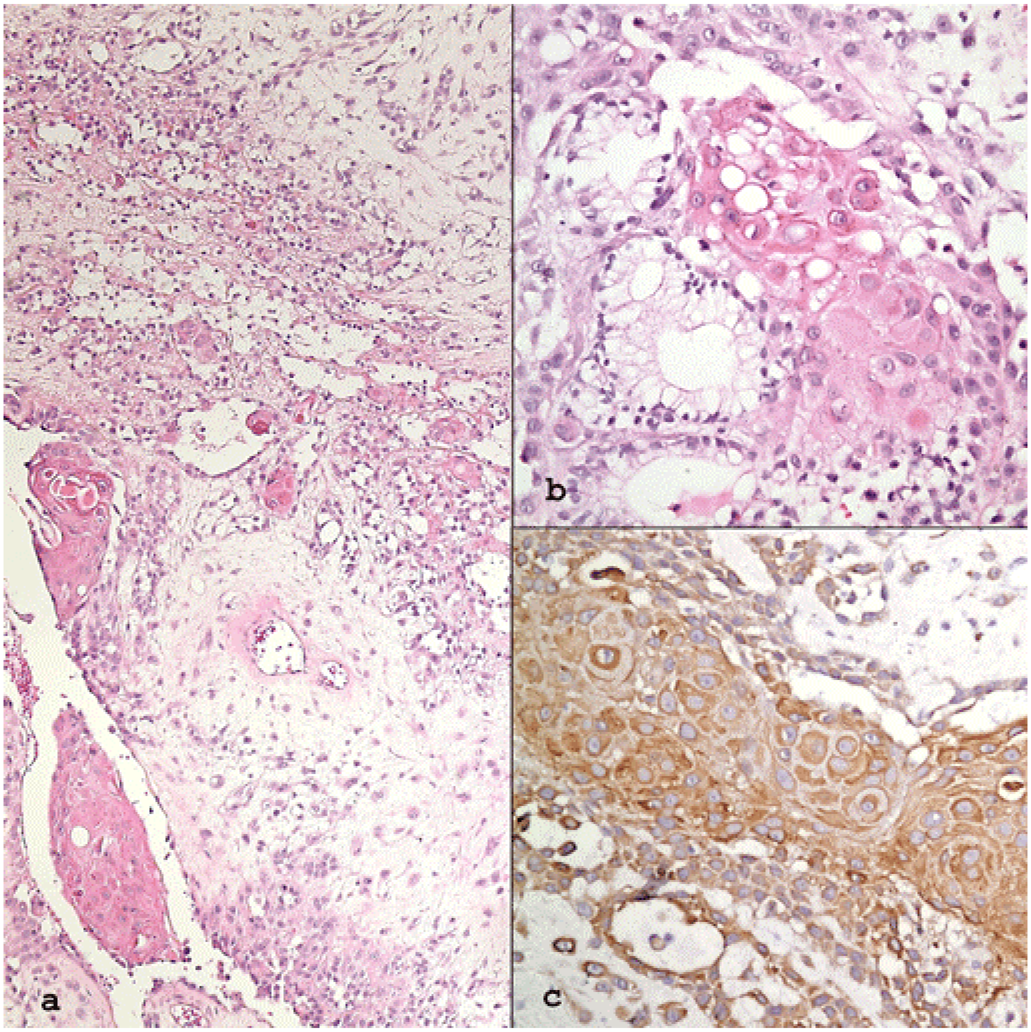

**FIGURE 1.** (a) Adenosquamous carcinoma with sarcomatous transformation (H-E ×100); (b) adenoid and squamous elements side by side (H-E ×200); (c) both glandular and squamous components express PSA immunohistochemically (immunohistochemistry, anti-PSA Ab ×200).

4.8- × 3.5-cm mass in prostatic fossa, infiltrating the base of the bladder. Seminal vesicles were not differentiated from mass and inguinal metastatic lymph nodes were present (largest 3.8 cm). Bone scans identified metastases in vertebrae, ribs, and pelvis. CT showed metastatic nodules in lung. The patient was experiencing severe back and pelvic pain. He received hormonal treatment with total androgen blockage, and external beam radiation to metastatic bone lesions and to whole pelvis. Vertebral areas C6-T1 and T12-L2 were delivered a total dose of 3000 cGy for each; pelvis was administered 4000 cGy totally. Then ten courses of taxol were given (75 mg/m$^2$ every 3 weeks). Serum PSA dropped to 0.27 ng/ml. However, pelvic CT 1 year after diagnosis revealed increase in the diameter of the prostate to 7 cm and an additional metastatic lymph node in mesorectal fascia. Six months later, the patient complained of difficulty in voiding. Physical examination showed hydroceles on both sides. No mass was detected on palpation of the testicles. TURP and bilateral orchiectomy were performed. Microscopy of TURP identified the tumor with the same morphology. Left testis had 2.5- × 1.5- × 1.5-cm metastatic mass (Fig. 2). It consisted of only glandular elements with no squamous or sarcomatoid component microscopically. Immunohistochemistry confirmed prostatic origin with apical PSA and diffuse prostate specific acid phosphatase (PSAP) positivity.

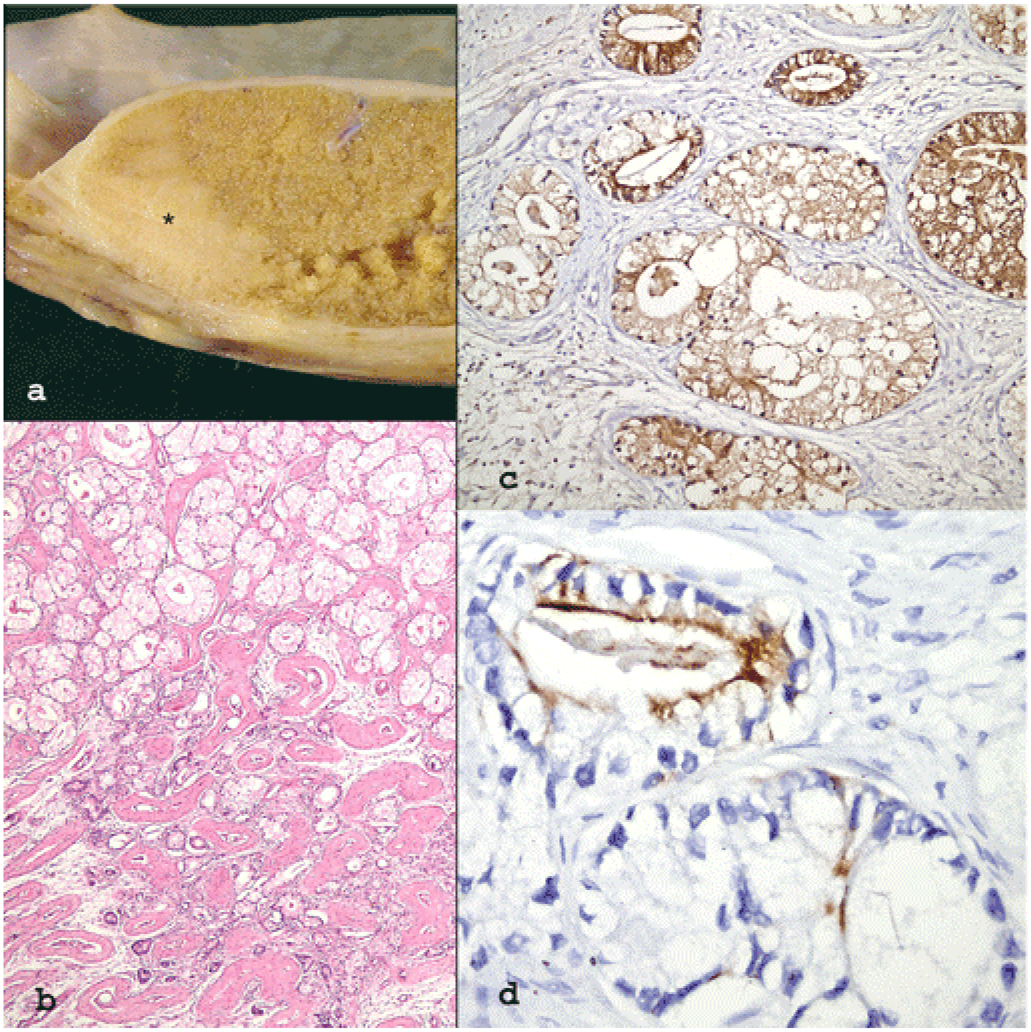

**FIGURE 2.** (a) Metastatic tumor in testis appears as irregular white focus, indicated by *; (b) neoplastic glands with foamy cytoplasm infiltrate between seminiferous tubules (H-E ×40); (c and d) they are positive diffusely for PSAP, focally for PSA (c: immunohistochemistry, anti- PSAP Ab ×200; d: immunohistochemistry, anti-PSA Ab ×400).

## DISCUSSION

Adenosquamous carcinoma of the prostate is a rare subtype characterized by coexistence of malignant glandular and squamous elements. They usually occur after hormonal or radiotherapy although de novo occurrence is also seen. Parwani et al. reported the largest series[4]. They commonly localize in transitional zone causing urinary obstruction, as in our case, and may present with metastatic disease[6,7]. Similarly, primary carcinosarcoma of the prostate is a rare and aggressive tumor. Epithelial component is mostly adenocarcinoma; urothelial and squamous types were also reported[8]. Adenosquamous morphology as the epithelial component in carcinosarcoma of prostate was first reported by Martin et al.[3]. Parwani described six cases of adenosquamous carcinoma with sarcomatoid transformation. In his series, only one was de novo like ours, others arose after radiation and/or hormone therapy. While the most frequent sites of metastasis are bone and lung, our case is the only one with documented testis metastasis.

Metastatic deposits from adenosquamous carcinoma may contain either glandular or squamous areas, or both. In our patient, it was only the glandular component. It exhibited abundant foamy cytoplasm and small dark nuclei. Foamy glands were also present in the primary in a limited extent. The most frequent sites for the metastasis from prostate cancer are bone, lymph nodes, and lung. However, among malignancies metastasizing to testis, prostate is the most common organ of origin. This probably reflects the frequent use of orchiectomy for hormone deprivation in the advanced disease. The route of spread is claimed to be through the lumen of the vas deferens or the lymphatics[9,10,11].

The cell of origin of adenosquamous carcinoma has been the subject of debate. In cases in which the tumor develops after courses of hormonal or radiotherapy in an initial pure adenocarcinoma, it is possible that treatment induces squamous metaplasia. Some authors have suggested the collision type tumor or divergent differentiation from an epithelial stem cell when the disease develops de novo. PSA staining of both squamous and glandular components in our case supports a common histogenesis from prostate secretory epithelium.

Among six such tumors in the series of Parwani, four with follow-up information survived only 16 months as mean life expanse. Our case is not an exception to the poor prognosis by these tumors. Although serum PSA dropped to low levels after hormonoradiotherapy, the size of prostate continued to increase and a new metastatic node appeared within 18 months.
